# Estimation of the Yield and Plant Height of Winter Wheat Using UAV-Based Hyperspectral Images

**DOI:** 10.3390/s20041231

**Published:** 2020-02-24

**Authors:** Huilin Tao, Haikuan Feng, Liangji Xu, Mengke Miao, Guijun Yang, Xiaodong Yang, Lingling Fan

**Affiliations:** 1 Key Laboratory of Quantitative Remote Sensing in Agriculture, Ministry of Agriculture and Rural Affairs, China, Beijing Research Center for Information Technology in Agriculture, Beijing 100097, China; wangcc@nercita.org.cn (H.T.); mengkemiao17@163.com (M.M.); yanggj@nercita.org.cn (G.Y.); yangxd@nercita.org.cn (X.Y.); p17301156@stu.ahu.edu.cn (L.F.); 2 School of Geodesy and Geomatics, Anhui University of Science and Technology, Huainan 232001, China; ljxu@aust.edu.cn; 3 National Engineering Research Center for Information Technology in Agriculture, Beijing 100097, China; 4 Beijing Engineering Research Center for Agriculture Internet of Things, Beijing 100097, China

**Keywords:** regression technology, yield, hyperspectral image, extracted plant height H_CSM_, estimation model, winter wheat

## Abstract

Crop yield is related to national food security and economic performance, and it is therefore important to estimate this parameter quickly and accurately. In this work, we estimate the yield of winter wheat using the spectral indices (SIs), ground-measured plant height (H), and the plant height extracted from UAV-based hyperspectral images (H_CSM_) using three regression techniques, namely partial least squares regression (PLSR), an artificial neural network (ANN), and Random Forest (RF). The SIs, H, and H_CSM_ were used as input values, and then the PLSR, ANN, and RF were trained using regression techniques. The three different regression techniques were used for modeling and verification to test the stability of the yield estimation. The results showed that: (1) H_CSM_ is strongly correlated with H (*R^2^* = 0.97); (2) of the regression techniques, the best yield prediction was obtained using PLSR, followed closely by ANN, while RF had the worst prediction performance; and (3) the best prediction results were obtained using PLSR and training using a combination of the SIs and H_CSM_ as inputs (*R^2^* = 0.77, RMSE = 648.90 kg/ha, NRMSE = 10.63%). Therefore, it can be concluded that PLSR allows the accurate estimation of crop yield from hyperspectral remote sensing data, and the combination of the SIs and H_CSM_ allows the most accurate yield estimation. The results of this study indicate that the crop plant height extracted from UAV-based hyperspectral measurements can improve yield estimation, and that the comparative analysis of PLSR, ANN, and RF regression techniques can provide a reference for agricultural management.

## 1. Introduction

Winter wheat is one of the most widely cultivated food crops in the world. Due to its high economic and nutritional value, the study of winter wheat is particularly important. The timely and accurate prediction of crop yield is an urgent issue in precision agriculture, and is of great significance for the formulation of national food policies, price regulation, the development of rural economies, and foreign food trade. Such prediction also assists agricultural managers in decision-making [1,2]. Standardized winter-wheat breeding requires the growth monitoring and yield forecasting of many plants. However, traditional methods of measuring yield can cause damage to crops, are time- and energy-intensive, have a low temporal and spatial resolution, and cannot be applied to large areas [3,4].

All objects absorb and reflect electromagnetic waves of different wavelengths. Different objects exhibit different spectral characteristics, and therefore objects can be identified based on their reflectance spectra. Remote sensing technology is developing rapidly, and can be used to obtain accurate crop canopy reflectance spectra in a dynamic and fast way. In turn, these spectra can be used to infer physiological and biochemical characteristics of crop canopies. There are various ways to obtain remote sensing technology, such as ground platforms, unmanned aerial vehicles (UAVs), and satellite platforms. Ground platforms cannot obtain orthophotos, and the data acquisition process requires large amounts of manpower and material resources, and moreover can cause damage to crops. However, both satellite remote sensing technology and UAV remote sensing technology can be used to monitor crops in a non-destructive way. Satellite remote sensing technology can be used to obtain information over a large scale [5,6], however it suffers from problems such as mixed pixels, long observation period, and low spatial and temporal resolution [7,8,9]. Unlike satellite remote sensing technology, UAV remote sensing technology can be used to monitor crops at a regional scale. It has the characteristics of low cost, convenient operation, high efficiency, high spatial and temporal resolution, and flexible measurement. Consequently, UAV remote sensing technology has begun to be applied for the acquisition of agricultural environmental information and crop monitoring [10,11,12,13]. The UAV sensors used for this purpose are mostly multispectral and digital cameras [14,15]. Multispectral and digital cameras are cheaper and lighter than hyperspectral cameras. However, hyperspectral sensors can obtain more specific information about the physiological parameters of crops [16,17,18,19,20,21,22,23]. Crop information obtained from hyperspectral sensors has been used to establish yield prediction models [23]. Therefore, as an efficient and accurate means to estimate crop yield, UAV-based hyperspectral measurements have received increasing attention from agricultural researchers.

Remote sensing technology can be used to estimate crop yield in various ways. However, crop yield is mainly estimated using either physical or statistical models. Both kinds of model have advantages and disadvantages [24]. Physical models are usually divided into a process model and a parameter model. Physical models are highly mechanical and can simulate the growth of crops, however the simulation requires a large number of input parameters, such as canopy physiological and biochemical parameters, soil data, weather data, and crop variety information. For example, in a previous study, soil data, the content of different components in crop leaves, and a vegetation index were used evaluate rapeseed yield using a physical model [25]. However, the data which are required as input to physical models are often difficult to obtain, which limits the application of physical models. In contrast, statistical models are mainly based on the relationship between remote sensing information and crop yield [26]. The estimation of crop yield using statistical models is mainly divided into two categories: The first includes direct estimation based on a vegetation index, such as the analysis of crop spectral characteristics to construct an inversion model [27]; the construction of a linear estimation model using a vegetation index [28]; and the second includes regression techniques, such as those based on a vegetation index; a partial least squares regression (PLSR) method which is used to construct different vegetation indices and yield inversion models [29]; estimation based on different vegetation indices combined with the partial least squares (PLS) method [3]; and the combination of an artificial neural network (ANN) and a vegetation index [30]. Several types of regression methods have been used to estimate crop yield; for example, a previous study used a combination of PLS and (1) multiple linear regression (MLR) and (2) an ANN to predict the yield of citrus fruit trees, and found that the PLS-MLR model was more accurate and stable than the PLS-ANN model [31]. Regression techniques have achieved relatively good results in the prediction of crop yield. However, the potential of hyperspectral data to predict crop yield is relatively under-explored.

In previous research, most scholars only used vegetation indices or regression techniques to improve the accuracy of crop yield inversion. For instance, Zhao et al. [29] estimated soybean yield, obtaining an *R^2^* of 0.659, while Ye et al. [31] used PLS to invert the yield of citrus fruit trees, obtaining an *R^2^* of 0.75 for the best inversion model. These studies mainly focused on using vegetation indices as a modeling factor, however did not introduce new independent variables in the yield estimation models. The present study investigates the effects of new independent variables on the model-based estimation of crop yield. Specifically, for the first time, this study uses crop plant heights extracted from UAV-based hyperspectral images as independent input variables in a yield prediction model and uses multiple regression techniques to determine the optimal estimation model.

The main purpose of this study was to evaluate the accuracies of winter-wheat yield estimations based on the ground-measured plant height (H), the plant height extracted from UAV-based hyperspectral images (H_CSM_), and the spectral indices (SIs), using PLSR, ANN, and RF regression techniques. 

The remainder of this paper is organized as follows: Section 2 introduces the research area, the experimental design, the acquisition and processing of ground data and UAV-based hyperspectral remote sensing data, and the extraction of winter-wheat plant height, and additionally discusses the determination of the spectral indices, analysis methods, and statistical analysis; Section 3 introduces the results, including the yield-prediction ability of the extracted winter-wheat plant height, the measured plant height, and the spectral indices (SIs) both alone and in combination, as well as the yield-prediction abilities of the PLSR, ANN, and RF regression techniques; Section 4 presents a discussion; and Section 5 lists conclusions.

## 2. Materials and Methods

### 2.1. Research Area and Test Design

**Figure 1:**
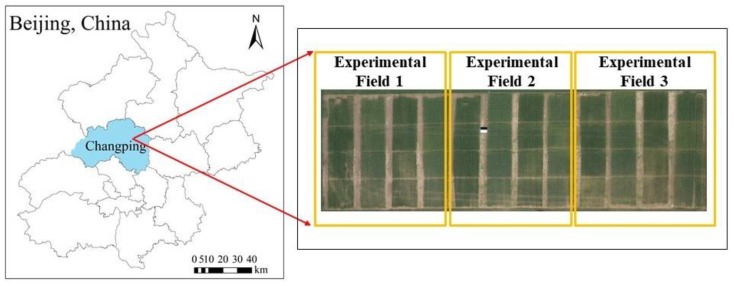
Location and aerial images of the test area.

The research area is located in the National Precision Agriculture Research Demonstration Base in Xiaotangshan Town, Changping District, Beijing, China, at 40°00′–40°21′ N, 116°34′–117°00′ E. The area lies on open terrain at the junction of the Wenyu River alluvial plain and Yanshan mountain. The research area has a warm temperate continental monsoon climate, with an average annual temperature of about 16 °C and an average annual rainfall of about 54 mm. Most precipitation occurs in summer. The geographical location of the research area and the UAV sampling sites are shown in Figure 1. There were a total of 48 test plots in the sampling area, and each test plot measured 8 m in length and 6 m in width. The planted winter-wheat varieties were Zhongmai 175 (ZM175) and Jing 9843 (J9843). The winter wheat was treated with nitrogen fertilization of 0 kg N/hm^2^ (N1), 195 kg N/hm^2^ (N2), 390 kg N/hm^2^ (N3), and 780 kg N/hm^2^ (N4), and was subjected to three moisture levels: rainfall only, normal water (100 mm), and twice normal water (200 mm); the water irrigation times were 20 October 2014, 09 April 2015, 30 April 2015, and 22 May 2015. 

### 2.2. Ground Data Acquisition and Processing 

Ground-based crop yield data and plant height data were obtained in the experimental plots at the winter-wheat jointing stage (21 April 2015), flagging stage (26 April 2015), and flowering stage (13 May 2015).

#### 2.2.1. Yield Acquisition

The crop yield was measured over the entire research area one day after the start of the winter-wheat mature period. Winter-wheat grain was collected from five sampling areas in each plot, each of which had an area of 1 m^2^; that is, grain was collected from a total area of 5 m^2^ in each plot. After sampling, GPS was used to determine the positions of the sampling areas to assist in the subsequent acquisition of UAV remote sensing data. The collected grain samples were transported to a laboratory where they were threshed, weighed, and assessed for quality. Then, 50 g of sample was placed in an envelope, which was then dried in an oven at 80 °C until a constant mass was obtained. Then, the sample was weighed again with a balance (accuracy of 0.001 g) and the water content of the sample was calculated based on the difference between the original weight and the dry matter mass. Finally, the winter-wheat yield was calculated based on the quality of the grain, the moisture content of the grain, and the sampling area, resulting in a total of 48 sets of yield data. 

#### 2.2.2. Measurement of Plant Height

A total of 20 winter-wheat plants were selected from the center of each plot. The height of each plant was then measured from the ground to the top using a ruler (accuracy of 0.5 mm), and the average height of the 20 plants was taken as the plant height for that plot.

### 2.3. Acquisition and Processing of UAV-Based Hyperspectral Remote Sensing Data

**Figure 2:**
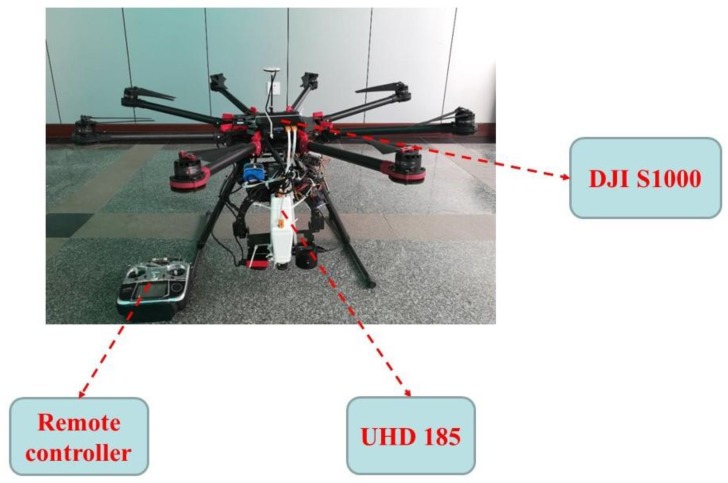
The unmanned aerial vehicle (UAV) remote sensing platform (model DJI S1000) that was used to acquire hyperspectral images. UHD 185: UHD 185 Firefly hyperspectral sensor.

An eight-rotor UAV remote sensing platform (DJI S1000 UAV, SZ DJI Technology Co., Ltd., Sham Chun, China; Figure 2) was used to acquire hyperspectral images of the experimental plots. The UAV has a single arm length of 386 mm, a net body weight of 4.2 kg, a carrier take-off mass of 6 kg, a flight height of 50 m, and two 18,000 mAh (25 V) batteries, which can last for 30 minutes flying at a speed of 8 m/s.

The UAV was equipped with a UHD 185 Firefly hyperspectral sensor (UHD 185 firefly, Cubert GmbH, Ulm, Baden-Württemberg, Germany). The sensor has a size of 195 × 67 × 60 mm^3^ and a weight of 0.47 kg, and has 125 spectral channels and a band range of 450–950 nm. The interval of each waveband is 4 nm. Five hyperspectral images were acquired per second, and the UHD185 was positioned vertically downward during the measurement process. In order to prevent effects due to shadow and wind, images were acquired on days with low wind speed and without significant cloud cover. Images were acquired at 12:00. The images were acquired on three flights on 21 April 2015, 26 April 2015, and 13 May 2015, corresponding to the winter-wheat jointing stage, flagging stage, and flowering stage, respectively. The spatial resolution of the hyperspectral images is 21 cm. After the hyperspectral image data were collected, and data were pre-processed. The pre-processing included two main parts: (i) Stitching and geometric correction of hyperspectral images following the method of Turner et al. [32]. This step was performed using the Cubert Cube-Pilot software (Cube-Pilot, version 1.4, Cubert GmbH, Ulm, Baden-Württemberg, Germany). Each hyperspectral image was fused to obtain a corresponding full-color image. After fusion, the Agisoft PhotoScan software (version 1.1.6; Agisoft LLC, St. Petersburg, Russia; hereinafter referred to as “PhotoScan”) was used to complete the stitching of the hyperspectral images. The spatial resolution of the stitched images is about 5 cm. Simultaneously, a digital surface model (DSM) was generated for the three growth stages of winter wheat (jointing, flagging, and flowering); (ii) The reflectance of the canopy spectrum was extracted. According to the spliced hyperspectral images, the area of each plot was observed, the ArcGIS software (version 10.2; Esri, Redlands, CA, USA) was used to draw the area vector of each plot, and the average spectrum of the vector region was used to calculate the average spectrum of the statistical vector region as the winter-wheat canopy spectrum in the different plots.

### 2.4. Extraction of Winter-Wheat Plant Height

In order to determine the winter-wheat plant height, a digital elevation model (DEM) of the experimental plots—which represents the elevation of the soil under the crops in the plot—was subtracted from the generated digital surface model (DSM)—which contains DEM and crop elevation information—in order to obtain a crop surface model (CSM), which was then used to extract the winter-wheat plant height. 

### 2.5. Spatial Map of Yield

A yield distribution map was constructed using the ENVI software (ITT Visual Information Solutions, Boulder, CO, USA). First, the UAV hyperspectral image was imported into ENVI, then the yield prediction model equation was input in Basic Tools, and finally the yield distribution map was generated.

### 2.6. Selection of Spectral Indices

In recent years, many researchers have shown that spectral indices are closely related to physiological and biochemical parameters of crops. Due to the application of hyperspectral observations in agricultural monitoring, many spectral indices are used to monitor crop growth. Therefore, based on the existing literature, we selected 20 spectral indices to estimate winter-wheat yield (Table 1). 

### 2.7. Regression Techniques

Partial least squares regression is a data analysis method which focuses on the characteristics of principal component analysis, typical correlation analysis, and linear regression analysis methods in the modeling process. However, since PLSR can effectively use spectral information, it has been widely used in the remote sensing of vegetation and has achieved good research results [51,52].

The basic principle of an ANN is to imitate the operation of the neurons in the human brain in order to simulate the way the brain processes information. The learning and training of neural networks requires a set of input data. An important ability of a neural network is that it can learn from the computing environment through the constant adjustment of its neuron weights and thresholds, and the network training is considered to be complete when the output error of the network reaches the expected value [53].

Random forest is a statistical data analysis method first proposed by Breiman and Cutler. Through bootstrapping sampling, the sample is returned to the sampling method for multiple sampling to form a training set [54]. The data to be predicted are classified by constructing a decision tree, and the category with the most occurrences is selected as the predicted category [55].

### 2.8. Statistical Analysis

Yield estimation models using PLSR, ANN, and RF were constructed using the MATLAB software (version, 2014a; MathWorks, Inc., Natick, MA, USA) on Microsoft Windows.

In order to build a yield prediction model, two-thirds of the data (n = 32, ground-measured plant height or yield) were selected as the modeling dataset, and the remaining one-third of the samples (n = 16, ground-measured plant height or yield) were used as the verification dataset at each winter-wheat growth stage. Three evaluation indicators were used to determine the accuracy of the yield prediction model, namely the coefficient of determination (*R^2^*), root-mean-square error (RMSE), and normalized root-mean-square error (NRMSE) [3]. The calculation equations for these parameters are given as follows:

(1) $$R^{2}=1-\frac{\sum_{i=1}^{n}{\left(x_{i}-y_{i}\right)}^{2}}{\sum_{i=1}^{n}{\left(x_{i}-\bar{x}\right)}^{2}}$$

(2) $$RMSE=\sqrt{\frac{\sum_{i=1}^{n}{\left(y_{i}-x_{i}\right)}^{2}}{n}}$$

(3) $$NRMSE=\frac{RMSE}{\bar{X}}$$

where $x_{i}$ is the measured plant height or plant yield for winter wheat, $\bar{x}$ is the average measured plant height or yield, $y_{i}$ is the plant height or yield predicted by the model, and *n* is the number of data points.

## 3. Results and Analysis

### 3.1. Extraction of Winter-Wheat Plant Height 

The ArcGIS software was used to extract the average plant height in each plot in the jointing stage, flagging stage, and flowering stage, respectively. 

The above was achieved using the following main steps:

(1) Point coordinate information was obtained using the ArcGIS software, and then the elevations of the soil points were extracted using the ArcTooLbox tool in the ArcGIS software;
(2) The DEM was generated based on the elevations of the soil points using the kriging tools in the ArcGIS software;
(3) The grid calculator tool in the ArcGIS software was used to extract the CSM of winter wheat by subtracting the DEM of the soil points from the DSM;
(4) Obtain the height of winter wheat plants in each plot by using the ROI tool in the ArcGIS software.

**Figure 3:**
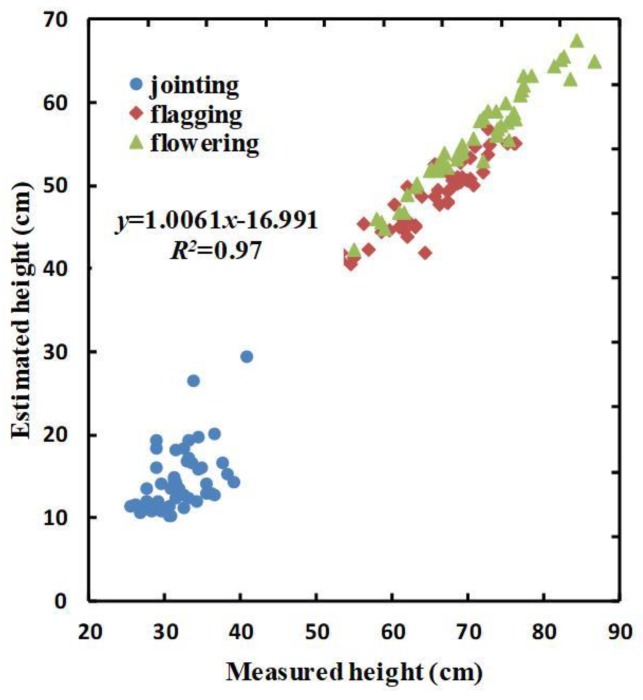
Measured and estimated heights of winter wheat. The three colors represent different growth stages: blue: jointing stage; red: flagging stage; green: flowering stage.

A total of 144 sets of average plant height data were obtained. Then, the extracted winter-wheat H_CSM_ was compared with the measured plant height. The results are shown in Figure 3.

From Figure 3, it can be seen that there is a strong correlation between the measured values of plant height and the values of H_CSM_ (*R^2^*= 0.97; y = 1.0061x − 16.991), although the H_CSM_ extracted during the three growth stages is less than the measured plant height. This suggests that the proposed model has a strong ability to predict winter-wheat plant height.

### 3.2. Relationship between Yield and Optimal Spectral Indices, H, and H_csm_

**Figure 4:**
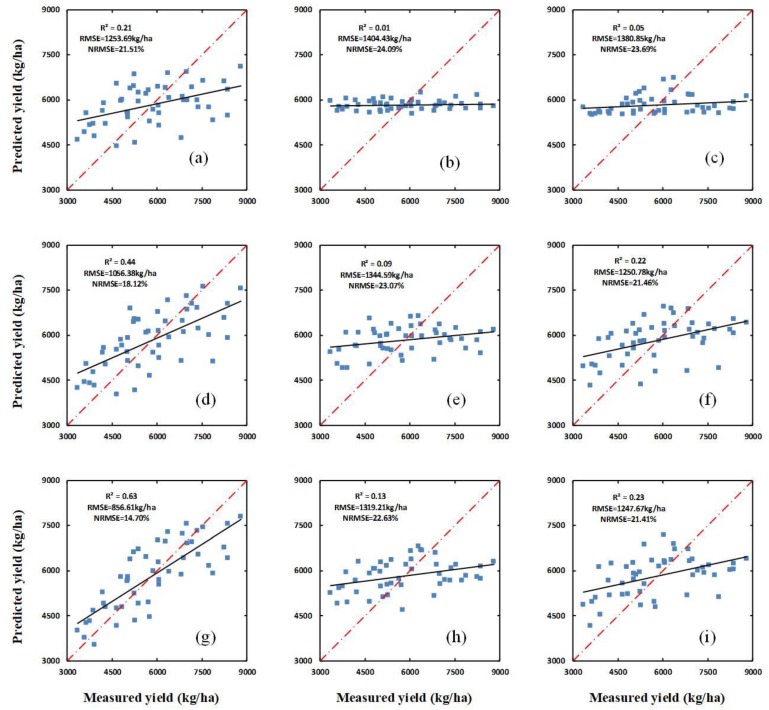
The relationships between the ground-measured winter-wheat yield and the yield predicted using the PBI, H, and H_CSM_ in different growth stages of winter wheat. (**a**–**c**) show the relationships between the measured yield and the yield predicted using the PBI, H, and H_CSM_ at the jointing stage, respectively; (**d**–**f**) show the same as (**a**–**c**) for the flagging stage; and (**g**–**i**) show the same as (**a**–**c**) for the flowering stage.

In order to study the relationships between yield and the optimal spectral indices (i.e., the PBI), H, and H_CSM_ at the jointing, flagging, and flowering stages of winter wheat, a linear regression method was performed. The results are shown in Table 2 and Figure 4.

As can be seen from Table 2 and Figure 4, the yield-prediction performance of the PBI, H, and H_CSM_ are different in each growth stage. At the jointing stage, the PBI performed poorly, with an *R^2^* of 0.21, while H and H_CSM_ both performed extremely poorly, with *R^2^* values of 0.01 and 0.05, respectively. During the flagging stage, the PBI, H, and H_CSM_ all performed better than during the jointing stage, with *R^2^* values of 0.44, 0.09, and 0.22, respectively. Similarly, in the flowering stage, the *R^2^* values for PBI, H, and H_CSM_ were all higher than in the flagging stage, with values of 0.63, 0.13, and 0.23, respectively. Meanwhile, the RMSE and NRMSE values for each of the three parameters all decrease over subsequent growth stages. All of the above results indicate that the yield-prediction performances of the PBI, H, and H_CSM_ all improve significantly between the jointing stage and the flagging stage. However, the performances of H_CSM_, and particularly of H, are significantly poorer than those of the PBI.

**Figure 5:**
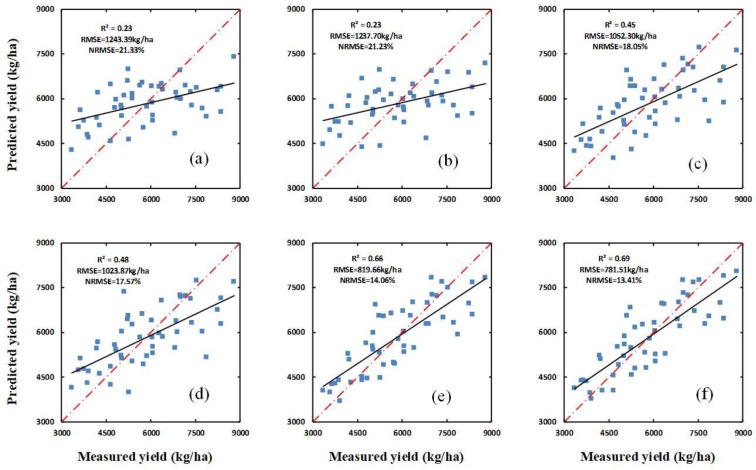
The relationships between the measured yield and the yield predicted using a combination of the PBI and H, and the yield predicted using a combination of the PBI and H_CSM_, at different growth stages (**a**,**b**) show the relationships between the measured yield and the yield predicted using a combination of the PBI and H, and using a combination of the PBI and H_CSM_, respectively, for the jointing stage; (**c**,**d**) show the same as (**a**,**b**) for the flagging stage, and (**e**,**f**) show the same as (**a**,**b**) for the flowering stage.

The optimal vegetation indices—namely, the PBI—was combined with H and H_CSM_ for regression analysis. The results, given in Table 3 and Figure 5, show that for all three growth stages the yield predictions obtained using a combination of the PBI and H and a combination of the PBI and H_CSM_ are superior to the predictions obtained using the PBI, H, and H_CSM_ individually (see Table 2). For the yield predictions obtained using a combination of the PBI and H, and a combination of the PBI and H_CSM_, the *R^2^* values increase, and the RMSE and NRMSE decrease. For the yield predictions obtained using a combination of the PBI and H, the *R^2^* values are 0.23, 0.45, and 0.66 in the jointing, flagging, and flowering stages, respectively; the RMSE values are 1243.39, 1052.30, and 819.66 kg/ha, respectively; and the NRMSE values are 21.33%, 18.05%, and 14.06%, respectively. Meanwhile, for the yield predictions obtained using a combination of the PBI and H_CSM_, the *R^2^* values are 0.23, 0.48, and 0.69 in the jointing, flagging, and flowering stages, respectively; the RMSE values are 1237.70, 1023.87, and 781.51 kg/ha, respectively; and the NRMSE values are 21.23%, 17.57%, and 13.41%, respectively.

### 3.3. Using H, H_csm_, and Spectral Indices in Combination with Either Partial Least Squares Regression, Random Forest, or an Artificial Neural Network to Estimate Winter-Wheat Yield

PLSR, ANN, and RF methods based all spectral indices (SIs) were used to estimate the winter-wheat yield in the jointing stage, flagging stage, and flowering stage. Table 4 shows the yield-estimation results obtained using PLSR regression for (1) the SIs, (2) a combination of the SIs and H, and (3) a combination of the SIs and H_CSM_, and Table 5 and Table 6 show the same as Table 4 except for ANN and RF, instead of PLSR, respectively. For the estimations obtained using the SIs, a combination of the SIs and H, and a combination of the SIs and H_CSM_, the best results were all obtained for the flowering stage for the PLSR, ANN, and RF methods; using PLSR and the SIs, a modeling *R^2^*, RMSE, and NRMSE of 0.75, 676.86 kg/ha, and 11.09% were obtained, respectively; using ANN and the SIs, a modeling *R^2^*, RMSE, and NRMSE of 0.71, 746.56 kg/ha, and 12.23% were obtained, respectively; and using RF and the SIs, a modeling *R^2^*, RMSE, and NRMSE of 0.31, 1148.67 kg/ha, and 18.81% were obtained, respectively. Using PLSR and a combination of the SIs and H, a modeling *R^2^*, RMSE, and NRMSE of 0.76, 659.93 kg/ha, and 10.81% were obtained, respectively; using ANN and a combination of the SIs and H, a modeling *R^2^*, RMSE, and NRMSE of 0.72, 728.99 kg/ha, and 11.94% were obtained, respectively; and using RF and a combination of the SIs and H, a modeling *R^2^*, RMSE, and NRMSE of 0.36, 1090.09 kg/ha, and 17.85% were obtained, respectively. Meanwhile, using PLSR and a combination of the SIs and H_CSM_, a modeling *R^2^*, RMSE, and NRMSE of 0.77, 648.90 kg/ha, and 10.63% were obtained, respectively; using ANN and a combination of the SIs and H_CSM_, a modeling *R^2^,* RMSE, and NRMSE of 0.74, 695.45 kg/ha, and 11.39% were obtained, respectively; and using RF and a combination of the SIs and H_CSM_, a modeling *R^2^*, RMSE, and NRMSE of 0.44, 1009.82 kg/ha, and 16.54% were obtained, respectively. That is, for the flowering stage, the yield-estimation results obtained using a combination of the SIs and H and a combination of SIs and H_CSM_ are superior to those obtained using only the SIs when using the PLSR, ANN, and RF models, respectively. The same is also true for the jointing and flagging stages. Furthermore, inferior yield prediction is obtained using the RF model compared to the PLSR or ANN models for all growth stages and all combinations of SIs, H, and H_CSM_.

**Figure 6:**
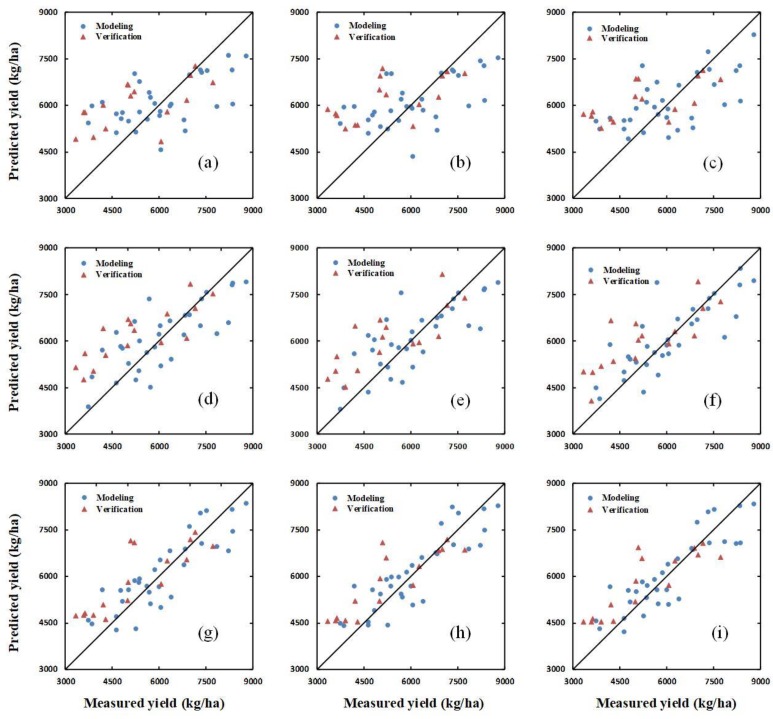
The relationships between the ground-measured and predicted values of winter-wheat yield at different growth stages based on partial least squares regression (PLSR). (**a**–**c**) show the results for SIs, a combination of SIs and H, and a combination of SIs and H_CSM_, respectively, at the jointing stage. (**d**–**f**) show the same as (**a**–**c**) for the flagging stage; and (**g**–**i**) show the same as (**a**–**c**) for the flowering stage.

**Figure 7:**
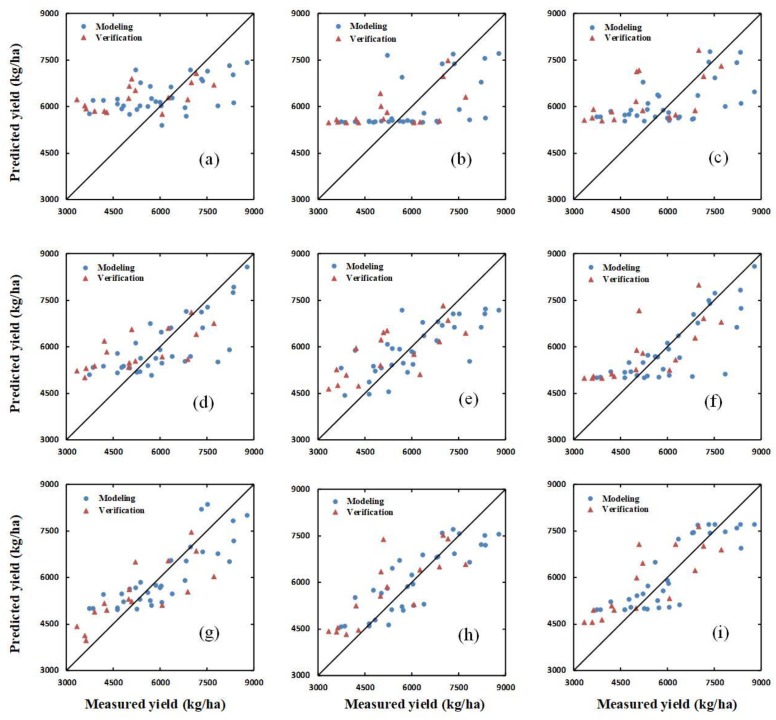
The relationships between the ground-measured and predicted values of winter-wheat yield at different growth stages based on an artificial neural network (ANN). (**a**–**c**) show the results for SIs, a combination of SIs and H, and a combination of SIs and H_CSM_, respectively, at the jointing stage. (**d**–**f**) show the same as (**a**–**c**) for the flagging stage; and (**g**–**i**) show the same as (**a**–**c**) for the flowering stage.

**Figure 8:**
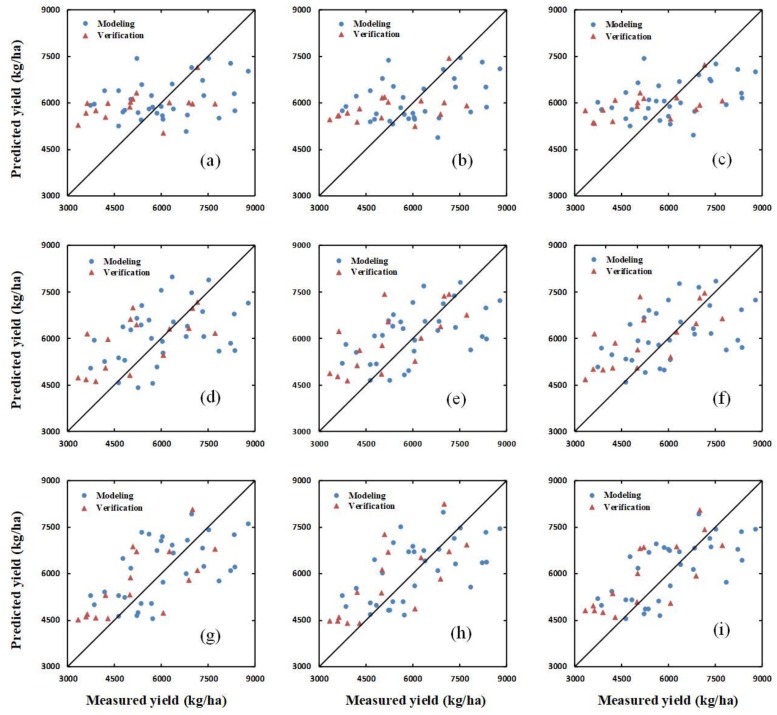
The relationships between the ground-measured and predicted values of winter-wheat yield at different growth stages based on the random forest (RF) method. (**a**–**c**) show the results for SIs, a combination of SIs and H, and a combination of SIs and H_CSM_, respectively, at the jointing stage. (**d**–**f**) show the same as (**a**–**c**) for the flagging stage; and (**g**–**i**) show the same as (**a**–**c**) for the flowering stage.

Additionally, the verification dataset was used to verify the yield-prediction ability of the SIs, a combination of SIs and H, and a combination of SIs and H_CSM_, using the PLSR, ANN, and RF methods, respectively. The results of the verification for PLSR, ANN, and RF are shown in Figure 6, Figure 7 and Figure 8, respectively.

### 3.4. Map of Predicted Yield

**Figure 9:**
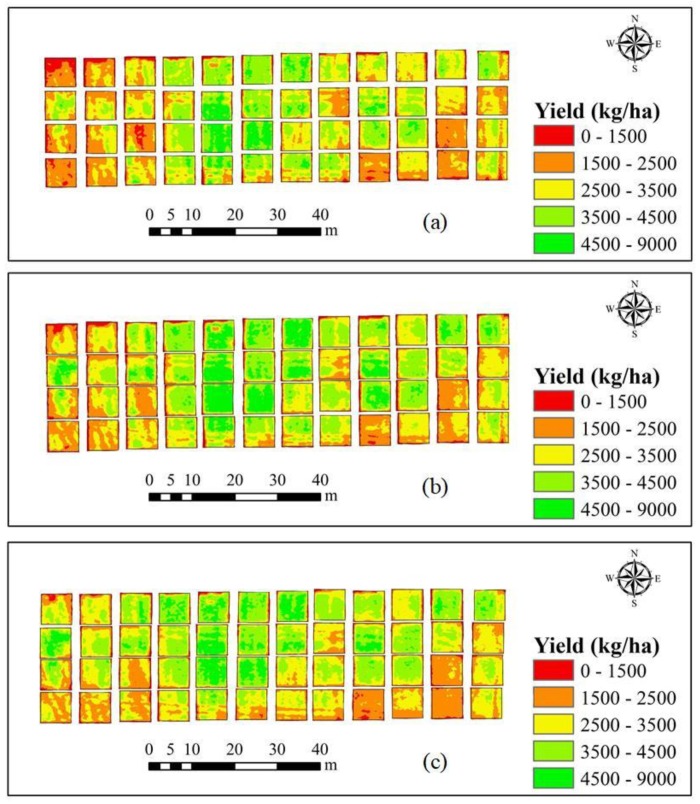
Maps showing the predicted yield of winter wheat in the 48 experimental plots obtained using PLSR and a combination of the SIs and H_CSM_. (**a**) Jointing stage, (**b**) flagging stage, and (**c**) flowering stage.

As can be seen from the results in Table 4, Table 5 and Table 6 and Figure 6, Figure 7 and Figure 8, the best yield prediction is obtained using the PLSR and a combination of the SIs and H_CSM_. Therefore, using this approach, the spatial distribution of winter wheat yield was estimated across the 48 experimental plots for each of the three winter-wheat growth stages, as shown in Figure 9a–c. As can be seen in the figure, the spatial distribution of predicted yield is similar for all growth stages and the range in predicted yield is 3500–9000 kg/ha. Additionally, the predicted yield distribution map shows that the yield of experimental field 2 is higher than that of experimental fields 1 and 3 for all three growth stages. 

## 4. Discussion

### 4.1. Estimation of Winter-Wheat Plant Height 

Extracting the plant height of crops from remote sensing data is of great significance for the management of agricultural production [56,57,58] and can help improve the efficiency of agricultural management. In this paper, a total of 144 manual measurements of plant height obtained during the three main growth stages of winter wheat (i.e., 48 samples per growth stage) were used to analyze the relationship between H_CSM_ and H. The *R^2^* was 0.97 (Figure 3), indicating that the extracted plant height can be reasonably used to estimate the actual plant height. This finding is consistent with the research results of Bendig et al. [57]. However, the value of H_CSM_ was generally smaller than that of H. In this study, UAV-based remote sensing hyperspectral reflectance data were obtained for the winter-wheat canopy in the experimental plots. Since the measured plant height is the height of the highest point of the leaves under growth conditions, H_CSM_ is generally lower than H.

### 4.2. Yield Estimation Using Spectral Indices, H, and H_csm_

Plant height can reflect the growth status of crops [59], and can also be used to estimate the LAI and biomass of crops [56,57], both of which are closely related to crop yield. The results of this study show that the yield of winter wheat can be estimated using a combination of a single optimal spectral index (in this case, the PBI) and H_CSM_, but that yield estimation is poor when using only H or H_CSM_ (see Table 2 and Figure 4). The accuracy of the yield estimation obtained using only H_CSM_ (i.e., the estimation which does not consider PBI) does not improve by a large amount between the flagging stage and the flowering stage, which may be related to the fact that the canopy image obtained in the flowering stage contains more mixed pixels. The yield estimations obtained using a combination of the all spectral indices (SIs) and H, and a combination of the SIs and H_CSM_, respectively, were both superior to the estimation obtained using only the PBI for all three growth stages (Table 3 and Table 4, Figure 5 and Figure 6). Yue et al. [60] found that using multiple spectral indices to estimate crop parameters is superior to using a single spectral index. This finding is consistent with the present research results. Moreover, the yield estimations obtained using a combination of the SIs and H_CSM_ were superior to those obtained using a combination of the SIs and H for all growth stages. This can be attributed to the fact that H_CSM_ was obtained using hyperspectral measurements of the winter-wheat canopy, while H was obtained based only on ground data, which does not contain information about the crop canopy. Therefore, compared with H, H_CSM_ is more objective. The results also show that the yield prediction improves from the jointing stage to the flowering stage. This can be attributed to the fact that, as the crop height increases, the winter wheat canopy coverage increases, and the range of plant LAI, biomass, pigment content, and water content changes; therefore, since the SIs are related to these physiological parameters, the correlation between the SIs and yield increases over subsequent growth stages (Figure 4). Thus, the optimization of model independent variables is crucial for estimating plant yield based on remote sensing data.

### 4.3. Yield Estimation Using Partial Least Squares Regression, Random Forest, or an Artificial Neural Network 

Relatively accurate yield prediction was obtained using both PLSR and ANN, respectively (Table 4 and Table 5, Figure 7 and Figure 8), with PLSR obtaining marginally better results. In contrast, the prediction obtained using RF is relatively poor (Table 6). The superior prediction performance of PLSR and ANN was achieved for both the modeling results and the verification results (Table 4 and Table 5, Figure 7 and Figure 8). Atzberger et al. [61], Fu et al. [62], and Mirzaie et al. [63] found that PLSR can improve the accuracy of crop growth monitoring, which is consistent with the results of this study. Zhao et al. [29] and Ye et al. [31] estimated crop yield via modeling, obtaining maximum *R^2^* values of 0.659 and 0.75, respectively. In comparison, the best *R^2^* value obtained in this paper was 0.77. This shows that the yield estimation model constructed in this paper improves the estimation accuracy. The RF method is suitable for application to large datasets, which can explain its poor performance for crop-yield estimation in this study. The predicted yields for the three growth stages obtained using PLSR are relatively consistent with the ground-measured yield (see Table 4 and Figure 6). Ferencz et al. [64] and Franch et al. [65] monitored yield distribution using Landsat Thematic Mapper (TM) data and moderate Resolution Imaging Spectroradiometer (MODIS) data, respectively, both obtaining good results. Additionally, Kurtser et al. [66] found that RGB camera data can be used to accurately estimate yield. In future research, we plan to use different remote sensing data for yield estimation and to explore the estimation effect of various sensors. 

## 5. Conclusions

In the present study, UAV-based hyperspectral remote sensing images were used to estimate the yield of winter wheat. Spectral indices, ground-measured plant height (H), and plant height extracted from the hyperspectral remote sensing data (H_CSM_) were used to estimate the yield of winter wheat in three main growth stages using linear regression, PLSR, ANN, and RF methods. The following conclusions can be drawn:

(1) H_CSM_ is strongly correlated with H (*R^2^* = 0.97). This indicates that crop heights can be accurately estimated using remote sensing data, and that such data could therefore resolve the traditional lack of crop height monitoring. Therefore, the results of the present study validate the application of UAV-based hyperspectral remote sensing technology to agricultural management.
(2) The correlations between the ground-measured winter-wheat yield and the spectral indices (SIs), H, and H_CSM_ gradually increased over successive crop growth stages. Additionally, the yield estimation obtained using a combination of the SIs and H, or a combination of the SIs and H_CSM_, were superior to those obtained using the optimal spectral indices (PBI), H, or H_CSM_ alone.
(3) The PLSR and ANN methods can be used to estimate the yield of winter wheat with a relatively high accuracy, with the PLSR method allowing a slightly higher accuracy. However, the RF method is far less effective for estimating yield than PLSR or ANN.

Overall, the results of this study show that UAV-based hyperspectral images can be used to provide accurate estimates of winter-wheat yield, and could therefore be of great significance for guiding precision agricultural production.

## Figures and Tables

**Table 1:** The spectral indices used in this study.

Spectral Index	Equation	Reference
MSR	(R800/R760 − 1)/(R800/R670 + 1)^1/2^	[33]
NDVI	(R750 − R706)/(R750 + R706)	[34]
OSAVI	1.16 × (R800 − R670)/(R800 + R670 + 0.16)	[35]
PSND	(R800 − R470)/(R800 + R470)	[36]
NPCI	(R670 − R460)/(R670 + R460)	[37]
PBI	R810/R560	[38]
TVI	0.5 × [120(R750 − R550) – 200 × (R670 − R550)]	[39]
RDVI	(R800 − R670)/(R800 + R670)^1/2^	[40]
SPVI	0.4 × [3.7(R800 − R670) − 1.2 × |R530 − R670|]	[41]
SR	R750/R550	[42]
TCARI	3 × [(R700 − R670) − 0.2 × (R700 − R550)(R700/R670)]	[43]
MCARI	((R700 − R670) − 0.2 × (R700 − R550))(R700/R670)	[43]
EVI2	2.5 × (R800 − R670)/(R800 + 2.4 × R670 + 1)	[44]
PSSR	R800/R500	[36]
RARS	R760/R500	[45]
WDRVI	(0.1 × R800 − R670)/(0.1 × R800 + R670)	[46]
LCI	(R850 − R710)/(R850 + R670)^1/2^	[47]
PSRI	(R680 − R500)/R750	[48]
RVSI	[(R712 + R752)/2] − R732	[49]
GI	R554/R677	[50]

Note: MSR: modified simple ratio index; NDVI: normalized difference vegetation index; OSAVI: optimized soil adjusted vegetation index; PSND: pigment-specific normalized difference index; NPCI: normalized pigment chlorophyll ratio index; PBI: plant biochemical index; TVI: triangular vegetation index; RDVI: renormalized difference vegetation index; SPVI: spectral polygon vegetation index; SR: simple ratio vegetation index; TCARI: transformed chlorophyll absorption ratio index; MCARI: modified chlorophyll absorption ratio index; EVI2: two-band enhanced vegetation index; PSSR: pigment-specific simple ratio; RARS: ratio analysis of reflectance spectra; WDRVI: modified wide dynamic range vegetation index; LCI: linear combination index; PSRI: plant senescence reflectance index; RVSI: red-edge vegetation stress index; GI: greenness index.

**Table 2:** The statistical results of yield estimation obtained using the optimal SIs—that is, the plant biochemical index (PBI)—the ground-measured winter-wheat plant height (H), and the winter-wheat plant height extracted from UAV-based hyperspectral images (H_CSM_), respectively, for different growth stages.

Growth Stage	Parameter	Linear Regression		
		*R^2^*	RMSE (kg/ha)	NRMSE (%)
Jointing	PBI	0.21	1253.69	21.51
	H	0.01	1404.43	24.09
	H_CSM_	0.05	1380.85	23.69
Flagging	PBI	0.44	1056.38	18.12
	H	0.09	1344.59	23.07
	H_CSM_	0.22	1250.78	21.46
Flowering	PBI	0.63	856.61	14.70
	H	0.13	1319.21	22.63
	H_CSM_	0.23	1247.67	21.41

Note: *R^2^*: coefficient of determination; RMSE: root-mean-square error; NRMSE: normalized root-mean-square error.

**Table 3:** Results of yield estimation obtained using a combination of the PBI and H, and a combination of the PBI and H_CSM_, for different growth stages.

Growth Stage	Parameters	Linear Regression		
		*R^2^*	RMSE (kg/ha)	NRMSE (%)
Jointing	PBI+H	0.23	1243.39	21.33
	PBI+H_CSM_	0.23	1237.70	21.23
Flagging	PBI+H	0.45	1052.30	18.05
	PBI+H_CSM_	0.48	1023.87	17.57
Flowering	PBI+H	0.66	819.66	14.06
	PBI+H_CSM_	0.69	781.51	13.41

**Table 4:** Results of the estimation of the yield of winter wheat in different growth stages by using partial least squares regression (PLSR).

PLSR							
Growth Stage	Information	Modeling			Verification		
		*R^2^*	RMSE (kg/ha)	NRMSE (%)	*R^2^*	RMSE (kg/ha)	NRMSE (%)
Jointing	SIs	0.31	1118.26	18.32	0.35	1415.64	26.83
	SIs+H	0.32	1110.02	18.18	0.37	1364.44	25.86
	SIs+H_CSM_	0.40	1049.32	17.19	0.40	1287.33	24.40
Flagging	SIs	0.58	878.50	14.39	0.57	1155.72	21.91
	SIs+H	0.60	854.30	13.99	0.62	1102.19	20.89
	SIs+H_CSM_	0.66	786.30	12.88	0.65	1069.60	20.27
Flowering	SIs	0.75	676.86	11.09	0.70	989.44	18.75
	SIs+H	0.76	659.93	10.81	0.74	891.99	16.91
	SIs+H_CSM_	0.77	648.90	10.63	0.75	870.25	16.49

**Table 5:** Results of the estimation of the yield of winter wheat in different growth stages by using an artificial neural network (ANN).

ANN							
Growth Stage	Information	Modeling			Verification		
		*R^2^*	RMSE (kg/ha)	NRMSE (%)	*R^2^*	RMSE (kg/ha)	NRMSE (%)
Jointing	SIs	0.27	1191.73	19.52	0.34	1429.01	27.09
	SIs+H	0.28	1155.38	18.97	0.35	1385.63	26.26
	SIs+H_CSM_	0.35	1100.49	18.02	0.37	1347.29	25.54
Flagging	SIs	0.54	919.00	15.05	0.50	1197.21	22.69
	SIs+H	0.58	907.63	14.87	0.52	1182.43	22.49
	SIs+H_CSM_	0.63	842.73	13.80	0.56	1161.36	22.01
Flowering	SIs	0.71	746.56	12.23	0.64	1072.90	20.34
	SIs+H	0.72	728.99	11.94	0.67	1056.03	20.02
	SIs+H_CSM_	0.74	695.45	11.39	0.68	1048.66	19.88

**Table 6:** Results of the estimation of the yield of winter wheat in different growth stages by using the random forest (RF) method.

RF							
Growth Stage	Information	Modeling			Verification		
		*R^2^*	RMSE (kg/ha)	NRMSE (%)	*R^2^*	RMSE (kg/ha)	NRMSE (%)
Jointing	SIs	0.08	1312.32	21.49	0.17	1569.45	29.75
	SIs+H	0.13	1276.37	20.91	0.23	1464.87	27.77
	SIs+H_CSM_	0.16	1241.14	20.33	0.28	1352.07	25.63
Flagging	SIs	0.16	1296.39	21.23	0.41	1232.04	23.35
	SIs+H	0.22	1214.64	19.89	0.46	1224.28	23.21
	SIs+H_CSM_	0.27	1166.36	19.10	0.48	1211.56	22.96
Flowering	SIs	0.31	1148.67	18.81	0.51	1190.47	22.56
	SIs+H	0.36	1090.09	17.85	0.55	1168.01	22.14
	SIs+H_CSM_	0.44	1009.82	16.54	0.59	1125.47	21.33
